# The complete chloroplast genome of *Akebia longeracemosa* (Lardizabalaceae)

**DOI:** 10.1080/23802359.2021.1884018

**Published:** 2021-03-11

**Authors:** Xiong Xiao, Huisheng Deng, Lingling Xu, Feng Wen, Xingjie Han, Liang Liao, Tongjian Li

**Affiliations:** College of Pharmacy and Life Sciences, Jiujiang University, Jiujiang, Jiangxi Province, People’s Republic of China

**Keywords:** Complete chloroplast genome, *Akebia longeracemosa*, Lardizabalaceae

## Abstract

The complete chloroplast genome of *Akebia longeracemosa*, a monoecious woody vine endemic to China, was determined. The total genome size is 158,020 bp, containing a large single copy region of 86,659 bp, a small single copy region of 19,059 bp, and a pair of inverted repeats of 26,151 bp. The chloroplast genome encodes 113 unique genes, including 79 protein-coding genes, 30 tRNA genes and 4 rRNA genes. Among them, fifteen genes have one intron each, and three genes contain two introns. The overall GC content is 38.7%, while the corresponding values of LSC, SSC, and IR regions are 37.1, 33.6, and 43.1%, respectively. Phylogenetic analysis showed that *A. longeracemosa* was closely related to *A. trifoliata*.

*Akebia longeracemosa* Matsumura (Lardizabalaceae) is a monoecious woody vine, distributed in the narrow regions of Hunan, Taiwan, Guangdong and Fujian province (Wu et al. 2001; Christenhusz 2012). Like other plants of genus *Akebia*, it produces edible fruits and has great potential to be domesticated into an alternative high-value crop (Li et al. 2010). The stems and leaves of *A. longeracemosa* are similar to those of *A. quinata*, whereas the inflorescences are similar to those of *A. trifoliata.* Morphological and molecular evidences suggest that *A. longeracemosa* may originate from hybridization between *A. quinata* and *A. trifoliata*. Chloroplast genes have been widely used in phylogenetic studies, which can provide strong evidences for the origin of hybrid species. However, *A. longeracemosa* is too closely related to *A. quinate* and *A. trifoliata*, and a few chloroplast genes could not differentiate the three species. As complete chloroplast genomes can offer valuable information for maternal identification, they have been widely used for phylogenetic studies of hybrid speciation in recent years (Carbonell-Caballero et al. 2015). Now, we reported and characterized the complete chloroplast genome sequence of *A. longeracemosa* based on the next-generation sequencing.

Fresh leaves were collected from a single individual planted in the Germplasm nursery of *Akebia* in Jiujiang University, and the resource was gathered from Shaoguan, Guangdong province, China (25.19.603 N, 113.00.730E). The total genomic DNA was extracted using the modified CTAB method (Doyle 1987). The genomic library was sequenced on an illumine Hiseq X Ten platform with paired-end reads of 150 bp. The chloroplast contigs were obtained by a BLAST search using the *Akebia trifoliata* chloroplast as a reference (GenBank accession NC_029427). The obtained contigs were ordered and oriented by being aligned to the reference genome via BBMap (Bushnell 2014) implemented in Geneious (Kearse et al. 2012). The finished chloroplast genome was annotated using the Geneious ‘Annotate from Database’ tool (Kearse et al. 2012). Finally, the annotated complete chloroplast genome of *A. longeracemosa* was submitted to GenBank (accession MW009065).

The size of complete chloroplast genome of *A. longeracemosa* is 158,020 bp with high coverage (mean 1721×). It has a typical quadripartite structure, including a large single copy (LSC) region of 86,659 bp, a small single copy region (SSC) of 19,059 bp, and a pair of inverted repeats (IRs) of 26,151 bp. There are 79 protein coding genes, 30 tRNA genes, and 4 rRNA genes, consistent with the chloroplast genome of *A. trifoliata*. Among these genes, 15 genes (*atpF*, *ndhA*, *ndhB*, *petB*, *petD*, *rpl2*, *rpl16*, *rpoC1*, *rps16*, *trnA-UGC*, *trnG-UCC*, *trnI-GAU*, *trnK-UUU*, *trnL-UAA* and *trnV-UAC*) have one intron each, and three genes (*clpP*, *rps12* and *ycf3*) contain two introns. The overall GC content is 38.7%, while the GC content of LSC, SSC, and IR regions are 37.1, 33.6, and 43.1%, respectively.

To confirm the maternal parent and phylogenetic position of *A. longeracemosa*, the phylogenetic tree including *A. longeracemosa*, five Lardizabalaceae species, and the outgroup *Euptelea pleiosperma* was constructed by complete chloroplast genomes. The seven complete chloroplast sequences were aligned by MAFFT V7.309 (Katoh and Standley 2013). And the phylogenetic tree was conducted by MrBayes (Huelsenbeck and Ronquist 2001). Phylogenetic analysis results strongly supported that *A. longeracemosa* is most closely related to *A. trifoliata* (Figure 1). The chloroplast genome of *A. longeracemosa* will provide useful data to determine the speciation and phylogenetic position of *A. longeracemosa*.

**Figure 1.**
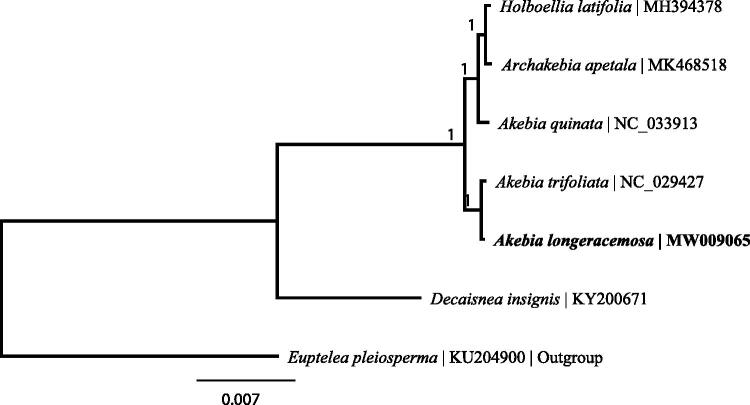
Bayesian inference (BI) tree based on the complete chloroplast genome sequences of 7 species. The numbers on the branches are posterior probability.

## Data Availability

The data that support the findings of this study are openly available in GenBank of NCBI at https://www.ncbi.nlm.nih.gov, reference number MW009065. Raw sequencing reads used in this study deposited in the NCBI Sequence Read Archive (SRA, http://www.ncbi.nlm.nih.gov/Traces/sra) with accession number PRJNA668399.
